# Hemozoin activates the innate immune system and reduces *Plasmodium berghei* infection in *Anopheles gambiae*

**DOI:** 10.1186/s13071-014-0619-y

**Published:** 2015-01-08

**Authors:** Maria L Simões, Luzia Gonçalves, Henrique Silveira

**Affiliations:** UEI Parasitologia Médica, Centro de Malária e Outras Doenças Tropicais, Instituto de Higiene e Medicina Tropical, Universidade Nova de Lisboa, Lisboa, Portugal; UEI Saúde Internacional e Bioestatística, Instituto de Higiene e Medicina Tropical, Universidade Nova de Lisboa, Lisboa, Portugal; CEAUL, Centro de Estatística e Aplicações da Universidade de Lisboa, Lisboa, Portugal

**Keywords:** *Anopheles*, *Plasmodium*, Hemozoin, Innate immunity, Parasite-vector interaction, REL2

## Abstract

**Background:**

Malaria is a worldwide infectious disease caused by *Plasmodium* parasites and transmitted by female *Anopheles* mosquitoes. The malaria vector mosquito *Anopheles* can trigger effective mechanisms to control completion of the *Plasmodium* lifecycle; the mosquito immune response to the parasite involves several pathways which are not yet well characterized. *Plasmodium* metabolite hemozoin has emerged as a potent immunostimulator of mammalian tissues. In this study, we aim to investigate the role of this parasite’s by-product as stimulator of *Anopheles gambiae* immunity to *Plasmodium berghei*.

**Methods:**

Female mosquitoes were inoculated with hemozoin and the *Plasmodium* infection rate and intensity were measured. Differences between treatments were detected by Zero-inflated models. Microarray transcription analysis was performed to assess gene expression response to hemozoin. Genome-wide analysis results were confirmed by stimulation of *Anopheles gambiae* tissues and cells with hemozoin and silencing of REL2-F and its negative regulator Caspar.

**Results:**

Gene expression profiles revealed that hemozoin activates several immunity genes, including pattern recognition receptors (PRRs) and antimicrobial peptides (AMPs). Importantly, we found that the Immune deficiency (Imd) pathway Nuclear Factor-kappaB (NF-κB) transcription factor REL2, in its full-length form REL2-F, was induced upon hemozoin treatment.

**Conclusions:**

We have for the first time shown the impact of hemozoin treatment in *Plasmodium* infection, reducing both rate and intensity of the infection. We propose that hemozoin boosts the innate immunity in *Anopheles*, activating key effector genes involved in mosquito resistance to *Plasmodium,* and this activation is REL2-mediated.

**Electronic supplementary material:**

The online version of this article (doi:10.1186/s13071-014-0619-y) contains supplementary material, which is available to authorized users.

## Background

The *Plasmodium* metabolite hemozoin is a by-product of the parasite’s digestion of host hemoglobin within the erythrocyte. Hemozoin is structurally similar to β-hematin, which is composed of cyclic heme dimers (Fe^III^-protoporphyrin IX). Heme dimers interact through hydrogen bonds, forming hemozoin crystals. Hemozoin is released together with merozoites upon rupture of parasitized erythrocytes. It reaches high concentrations in the circulation, and is engulfed by macrophages, monocytes, neutrophils and other immune cells [1]. It has been suggested that free hemozoin activates the host innate immune system in mammals [2]. In fact, during the last decade it has emerged as a potent immunoactivator, both *in vitro* and *in vivo*, stimulating macrophages and dendritic cells to produce pro- and anti-inflammatory cytokines and chemokines in mouse and human cells [2-4]. Although hemozoin’s pro-inflammatory role is generally accepted, the recognition and host response to this molecule, as well as the molecular mechanism(s) by which it activates the mammalian innate immune system, has been a subject of intense debate.

Coban *et al*. [5] first identified TLR9 (Toll-like receptor 9, the mammalian receptor for unmethylated CpG motifs in microbial DNA and synthetic oligonucleotides [6]), as mediator of the host response to hemozoin in a murine model. Parroche *et al*. [7] further reported that hemozoin is immunologically inert and that the activation of TLR9 is caused instead by hemozoin-conjugated parasite DNA. Recently, a study by Griffith and colleagues [8] showed that naïve dendritic cells or macrophages are not stimulated by hemozoin, but it augments the inflammatory response to malarial DNA or TLR ligands, supporting previous results [7]. Simultaneously, the work presented by Jaramillo *et al*. demonstrates that hemozoin possesses immunostimulatory properties by itself and it is unlikely that its immune activity is caused by the ability to bind parasitic DNA [3], in contrast with mentioned results [7,8]. A more recent study by Wu *et al*. [9] affirms that hemozoin is neither stimulatory by itself for activation of mouse and human dendritic cells, nor is it able to confer activity to parasite DNA. Their data also show that hemozoin is not a TLR9 ligand; indeed they are not the first suggesting that TLR9 has no role to play during malaria infection in both humans and mice [10,11]. Similar to the hemozoin’s TLR9 activation of the immune system, the activation through the NLRP3 (NLR family, pyrin domain containing 3) inflammasome is also being extensively debated [8,12].

In contrast to the number of studies regarding hemozoin’s immunity activation in mammals, one single study exploring the effect of this molecule in the *Anopheles* vector has been published so far [13]. The authors of the mentioned study [13] demonstrated that both *Plasmodium falciparum* and synthetic (sHz) forms of hemozoin contribute to the immune activation in *Anopheles*. In accordance with similar observations in mammalian cells [14], hemozoin was found to induce nitric oxide synthase expression in mosquito cells and tissues via multiple signalling pathways. In the mosquito, hemozoin is released from parasitized erythrocytes and leukocytes which enter the midgut with the infected blood meal [13]. *Anopheles gambiae* (*A. gambiae*)’s peritrophic matrix, acting as a barrier between the ingested blood and the midgut epithelium, is completely formed 24 h after a blood meal [15], thus the midgut epithelium is possibly exposed to hemozoin before the matrix is fully formed. Interactions of the peritrophic matrix with heme have been reported on several mosquito species and most recently Magalhaes [16] has reviewed and shown heme/iron-containing aggregates associated to the *A. gambiae* peritrophic matrix.

Given the challenge of finding effective ways to reduce the burden caused by malaria, a wealth of knowledge on the parasite-vector interaction and the mosquito immunity to *Plasmodium* has been building up in the last decade. Analysis of literature shows that the mosquito immune response to hemozoin is not yet well characterized and requires further elucidation. To address this issue, in this study we unravel the mechanism of action of hemozoin as stimulator of the mosquito immunity. We show for the first time the impact of hemozoin on the malaria vector resistance to the parasite, successfully impairing both rate and intensity of the infection. Furthermore, we observed that hemozoin activates the transcription of several key immune genes and that REL2-F transcription factor, rather than other factors, is induced by hemozoin *in vivo* and *in vitro.* These findings contribute to understanding the mosquito protective immune response mechanisms and establish hemozoin as a modulator of mosquito immunity, inducing the transcription of multiple effector genes that help the vector fight *Plasmodium* infection.

## Methods

### Ethics statement

The maintenance and care of experimental animals was carried out in strict accordance with the recommendations in the Europe Directive 86/609/EEC and Portuguese law (Decreto-Lei 129/92) for biomedical research involving animals, and was approved by the Divisão Geral de Veterinária (DGV), Portugal. All experiments were performed under anaesthesia, and all efforts were made to minimize animal suffering.

### Synthetic Hemozoin (sHz) preparation

sHz was prepared from high-purity hemin chloride, using a protocol as described in [3,12]. Briefly, 500 mg hemin (≥98% pure, Sigma) were dissolved in degassed NaOH (0.1 M, 100 ml) and pH adjusted with propionic acid. The mixture was heated at 70°C for 18 h. After cooling, the solid was separated and washed with three alternate washes of NaHCO_3_ and MilliQ water for 3 h. MeOH was then alternated with MilliQ water for three final washes. The sample was dried in a vacuum chamber overnight. The pigment was resuspended in endotoxin-free PBS at a final concentration of 2.5 mg/ml and kept at −20°C until further use.

### Mosquito treatment and infection

*A. gambiae* s.s. mosquitoes (Yaoundé strain) were reared and maintained as described previously [17]. Three to four day old female mosquitoes were cold-anaesthetized and inoculated intrathoracically with 69 nl of a 50, 100 or 200 μg/ml solution of sHz or with the same volume of endotoxin-free PBS, using a Nanoject micro-injector (Drummond Scientific). Mosquitoes were left to rest for 24 h. Female CD1 mice were intraperitoneally inoculated with 10^7^*Plasmodium berghei* (ANKA) GFPcon 259 cl2 parasitized red blood cells/ml and mosquitoes were fed as previously optimized in our Lab [17]. Four independent biological replicates were performed for each experiment. Between 8 and 10 days post-infection, mosquito midguts were collected to determine infection rate (prevalence) and intensity.

### Statistical methods to compare sHz-treated with PBS-treated mosquitoes

Following the descriptive statistics of all variables, the zero-inflated (ZI) models were explored taking into account the nature of the distribution of the number of oocysts. ZI models have been proposed to model count variables, dealing with an excess of zeros in several applications [18]. This type of model combines two components: one component is represented by a point mass at zero and another one is a count distribution such as Poisson or negative binomial. According to these distributions, the corresponding models are denoted by ZIP and ZINB. In parasitology, the number of eggs/oocysts tends to exhibit over-dispersion and/or an excess number of zeros and ZI models present advantages to classical statistical tests. Zero-inflated models can be fitted using R Program [19] through the function zeroinfl() of the pscl package [20]. The MASS package was also used to fit the well-known Generalized Linear Models (GLM) that typically are not sufficient for modelling excess zeros. The Voung test was used to verify if a GLM is indistinguishable from the corresponding ZI model. For fitting model, the log-likelihood value and Akaike Information Criterion (AIC) were obtained. Pearson residuals for different models were calculated to evaluate discrepancies between the observed number of oocysts and the expected number of oocysts predicted by each model.

### RNA isolation and Microarray hybridization

Female mosquitoes were dissected 24 h after sHz inoculation (immediately before feeding). Batches of *circa* 30 fat bodies (abdomen without midgut, ovaries and malpighian tubules, which can also comprise hemocytes that are in part sessile and attached to tissues) were dissected in cold DEPC treated PBS and processed for RNA preparation. Total RNA was prepared and concentration and purity determined as described before [17]. Each GeneChip experiment was performed with three biological replicates. RNA was processed for use on Affymetrix GeneChip *Plasmodium/Anopheles* Genome Arrays, according to the manufacturer’s One-Cycle Target Labeling Assay. Briefly, total RNA containing Poly-A RNA spiked controls (GeneChip Expression Eukaryotic Poly-A RNA Control Kit, Affymetrix) was used in a reverse transcription reaction to generate first-strand cDNA. After second-strand synthesis, double-stranded cDNA was used in an *in vitro* transcription (IVT) reaction to generate biotinylated cRNA (GeneChip Expression 3’-Amplification Reagents for IVT-Labeling, Affymetrix). Fragmented cRNA was used in a 300 μl hybridization containing added hybridization controls, on arrays for 16 h at 45°C. Standard post hybridization wash and double-stain protocols (EukGE-WS2v4) were used on an Affymetrix GeneChip Fluidics Station 450. Arrays were scanned on an Affymetrix GeneChip scanner 3000. All quality parameters for the arrays were confirmed to be in the recommended range.

**Figure 1:**
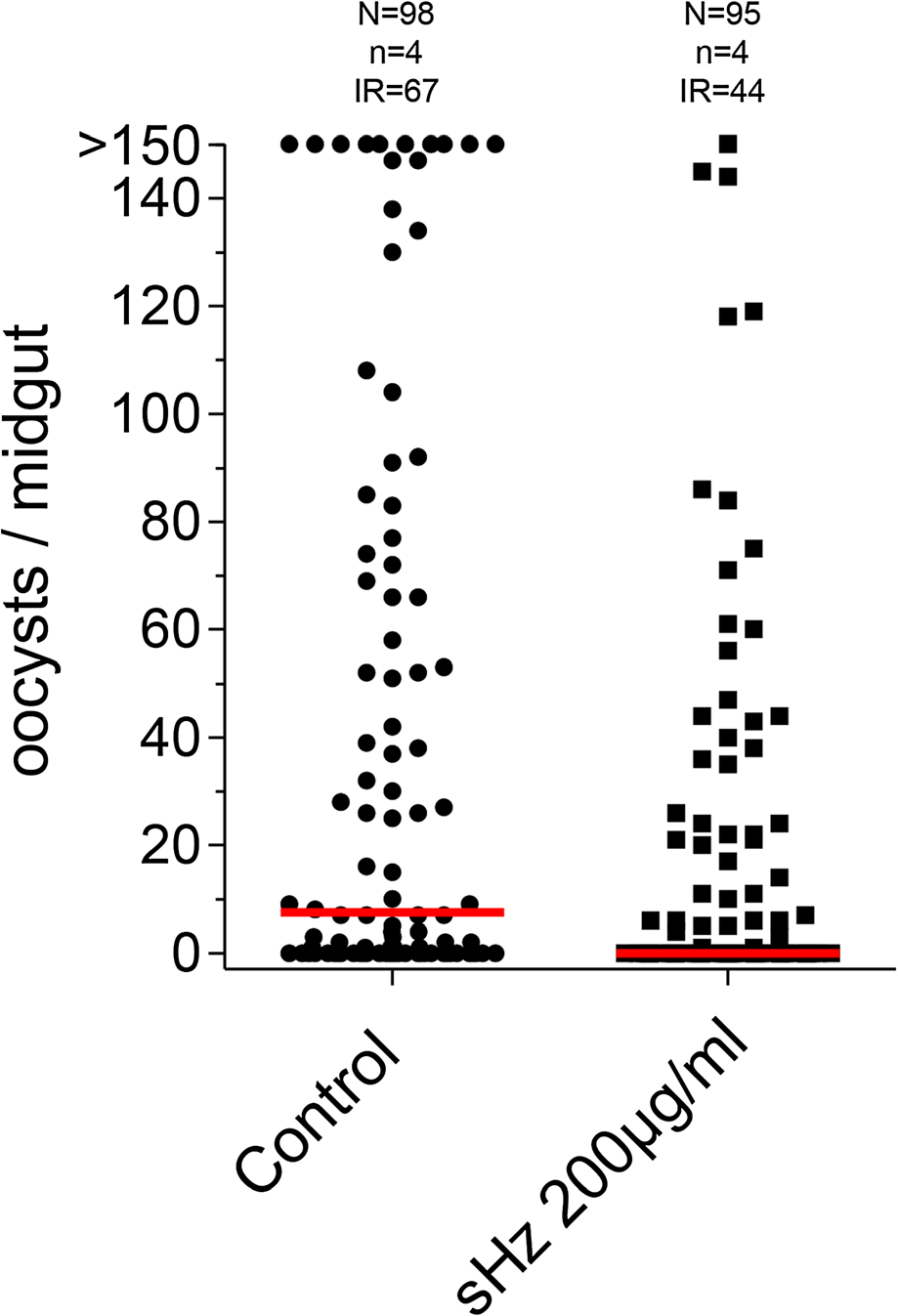
**Effect of hemozoin on** ***Anopheles gambiae*** **infection by** ***Plasmodium berghei*** **.** Female mosquitoes were treated with PBS-control or 200 μg/ml sHz and infected with *P. berghei* 24 h later. Oocysts were counted 8–10 days post-infection. Dots indicate the number of parasites in individual midguts, horizontal red bars represent the median. N, number of female mosquitoes; n, number of independent experiments; IR, infection rate (%).

A random batch of mosquitoes per treatment/per experiment was fed and left to check the success of infection (data not shown), confirming the pattern described in Figure 1.

### Microarray analysis

Scanned arrays were analysed first with Affymetrix MAS 5.0 software to obtain absent /present calls and for subsequent analysis with dChip 2006. The arrays were normalized to a baseline array with median CEL intensity by applying an Invariant Set Normalization Method [21]. Normalized CEL intensities of the arrays were used to obtain model-based gene expression indices based on a perfect match-only model [21]. Replicate data for the same sample type were weighted gene-wise by using inverse squared standard error as weights. Only genes called present in at least one of the arrays and within replicate arrays called present within a variation of 0 < median (standard deviation/mean) < 0.5 were kept for downstream analysis. All genes compared were considered to be differentially expressed if the 90% lower confidence bound of the fold change between experiment and baseline was above 1.2. The lower confidence bound criterion implies that we can be 90% confident that the fold change is a value between the lower confidence bound and a variable upper confidence bound. Li and Wong [21] have shown that the lower confidence bound is a conservative estimate of the fold change and therefore more reliable as a ranking statistic for changes in gene expression. Complete microarray data has been deposited in the ArrayExpress database [accession number E-MTAB-1072]. To validate the microarray data, the expression profiles of ten genes differentially expressed by microarray were analysed by qRT-PCR as described below: *AGAP000693, AGAP003689, AGAP005335, AGAP005848, AGAP006809, AGAP006911, AGAP010056, AGAP010812, AGAP011294* and *AGAP011790*. The sequences of primers used for amplification can be found in Additional file 1.

### Stimulation of *A. gambiae* cells

Immortalized *A. gambiae* hemocyte-like cells Sua 5.1^*^ were cultured as in [13] and incubated at 28°C. 1 to 1.5 × 10^6^ cells were seeded in 24-well plates and left to grow overnight. Cells were stimulated with different concentrations of sHz or endotoxin-free PBS as a control and incubated at 28°C for 24 or 48 h. Three independent experiments were performed.

### Tissue collection before and after blood ingestion

Mosquitoes were inoculated with 69 nl of a 200 μg/ml solution of sHz or with the same volume of endotoxin-free PBS, as described above. Batches of *circa* 30 mosquitoes were dissected 24 h after sHz inoculation (immediately before feeding) and fat bodies collected as before. The remaining mosquitoes were fed with either a *P. berghei*-infected or a noninfected (naïve) blood meal and left to rest overnight. 24 h after feeding, fat bodies from different groups were collected. Three independent experiments were performed.

### Real-time qRT-PCR analysis

Total RNA was isolated from mosquito cells and fat bodies using NucleoSpin RNA II kit (Macherey-Nagel), at 24 and 48 h after sHz treatment. Concentration and purity of RNA were determined by spectrophotometry. cDNA was synthetized using 1 μg of total RNA reverse transcribed with High Capacity RNA-to-cDNA Master Mix (Applied Biosystems). Quantitative analysis was performed by quantitative Real-time PCR using SYBR Green Supermix (Bio-Rad), on a final volume of 20 μl, using an iCycler iQ (Bio-Rad). One μl of cDNA was used as template. Cycle conditions were: an initial denaturation step at 95°C for 10 minutes, followed by 40 cycles at 95°C for 10 seconds and 62–65°C for 45 seconds. For all assays, the expression levels of target genes were normalized to the levels of ribosomal protein S7 gene (*AGAP010592*). The sequences of primers used for amplification can be found in Additional file 1.

### RNAi gene silencing

Specific dsRNA was synthesized using the MEGAscript T7 kit (Ambion), according to manufacturer’s instructions. Initial PCR products were generated from cDNA using gene specific primers (Additional file 1) that include a T7 promoter sequence. An exogenous gene, mouse *beta-2microglobulin (β2M)*, was used to produce control-dsRNA. Each PCR product was purified and dsRNA synthesized as in [17]. dsRNA concentration and quality were assessed by spectrometry and agarose gel.

#### Cells

Following results of a test-experiment done to assess the best *caspar*-dsRNA concentrations and silencing time-point (as explained in the results section), *A. gambiae* Sua 5.1^*^ cells were treated with 22.5 μg/ml *caspar-*targeting or *β2M*-targeting dsRNA (to serve as reference for silencing efficiency and for quantification of gene expression levels). One day following silencing, control-PBS or 12.5 μg/ml sHz was added to cells for 24 h. Total RNA was isolated from cells and cDNA prepared as described above, 48 h after dsRNA treatment (24 h post sHz adding). Efficiency of gene silencing and levels of differentially expressed genes in gene-silenced samples were assessed by qRT-PCR. For all assays, the expression levels of target genes were normalized to the levels of ribosomal protein S7 gene and compared to controls treated with dsRNA against *β2M*, by qRT-PCR using specific primers (Additional file 1). Three independent experiments were performed.

#### Mosquitoes

Three day old female mosquitoes were cold-anaesthetized and inoculated intrathoracically with 69 nl of a 3 μg/μl solution of dsRNA (207 ng) for each gene of interest. A control group was injected with dsβ2M (to serve as reference for silencing efficiency and for quantification of gene expression levels). All injections were performed using a Nanoject micro-injector (Drummond Scientific). Efficiency of gene silencing was assessed 4 days after dsRNA injection by collection of tissues followed by qRT-PCR. Silenced mosquitoes were treated either with control-PBS or 200 μg/ml sHz and the levels of differentially expressed genes in gene-silenced treated samples assessed 24 h later, before *P. berghei*-infected blood feeding. Infection rate and intensity were measured after 8 days. Three independent experiments were performed.

## Results

### Hemozoin impairs *Plasmodium berghei* infection in *Anopheles gambiae*

To define the impact of hemozoin treatment on the response of *A. gambiae* to *P. berghei* infection*,* mosquitoes were injected with high purity sHz, comparable to the one used in mice and other mosquito studies. This highly resembles the native *Plasmodium falciparum* hemozoin crystals in both size and physicochemical properties, and is devoid of protein and DNA contamination [3,13,22]. Independent experiments were performed using concentrations of 50 μg/ml, 100 μg/ml and 200 μg/ml. The lowest concentration treatment, 50 μg/ml, showed no differences when compared with the control (data not shown). A total of 221 *A. gambiae* females were treated with 100 μg/ml. Infection rate (number of infected mosquitoes per total number of mosquitoes observed) and intensity (number of oocysts per midgut) were consistently lower in sHz treated mosquitoes when compared to control, sterilized PBS treated ones (Additional file 2). When sHz concentration was increased to 200 μg/ml, the differences in the infection rate of sHz treated mosquitoes (44%) when compared to control mosquitoes (67%) were accentuated (Additional file 2). The number of oocysts per infected midgut was reduced, from 69 to 35, with 200 μg/ml sHz treatment, in a total of 193 *A. gambiae* females. Oocyst counts from the midguts of both infected and uninfected mosquitoes are shown in Figure 1. Reduction of both parameters was consistently observed in all replicate experiments (Additional file 2). Infection rate and intensity in the control group varied from 44.7% to 84.6% and 21.7 to 98.8 respectively (Additional file 2), which is within the normal range for this combination of *A. gambiae*/*P. berghei* strains.

In order to explore the possible effect of the experiments and the treatment, several zero-inflated models were tested. ZI models showed better performance than generalized linear models, pointing out the importance of modelling over-dispersion and/or an excess number of zeros in count variables such as the number of oocysts. On the other hand, models with negative binomial distribution (ZINB) revealed better fit than Poisson distribution (ZIP).

Estimated coefficients and p-values corresponding to the selected ZINB models for concentrations 100 μg/ml and 200 μg/ml showed that, for the lower concentration (100 μg/ml, n = 221), the treatment effect was not significant (at 5% significance level) for both components of the ZINB model (Additional file 3: Table S1). Comparing to experiment 1, some differences were found for experiments 2 and 3. For concentration 200 μg/ml (n = 193), the fitted model (Additional file 3: Table S2) revealed a significant effect of the treatment, with an increase of the non-infection rate and a reduction in the number of oocysts. In this case, the effect of the different experiments was not significant for both components. In general, the residual analysis showed a small magnitude (Percentile 75 was 0.1597–3Q in Additional file 3: Table S2). The values between 2 (approximately 1.96) and the maximum value (4.3472) represented 3.6% (of the 193 observations in study) and are related with the highest number of oocysts.

Taken together, these results show that hemozoin increases the mosquito resistance to infection by *Plasmodium*, through an impact on both infection rate and intensity. This suggests that pathways and effector mechanisms triggered by hemozoin are used by *A. gambiae* to control *P. berghei* infection. Although it is very unlikely that the observed effect of hemozoin is due to the inoculation of crystalline material into the hemocoel, we could not assess the contribution of the crystal-physical factor, as no appropriate control (inert crystal structures similar to hemozoin) is available that could be used in this study.

### Hemozoin stimulates the innate immune system

**Figure 2:**
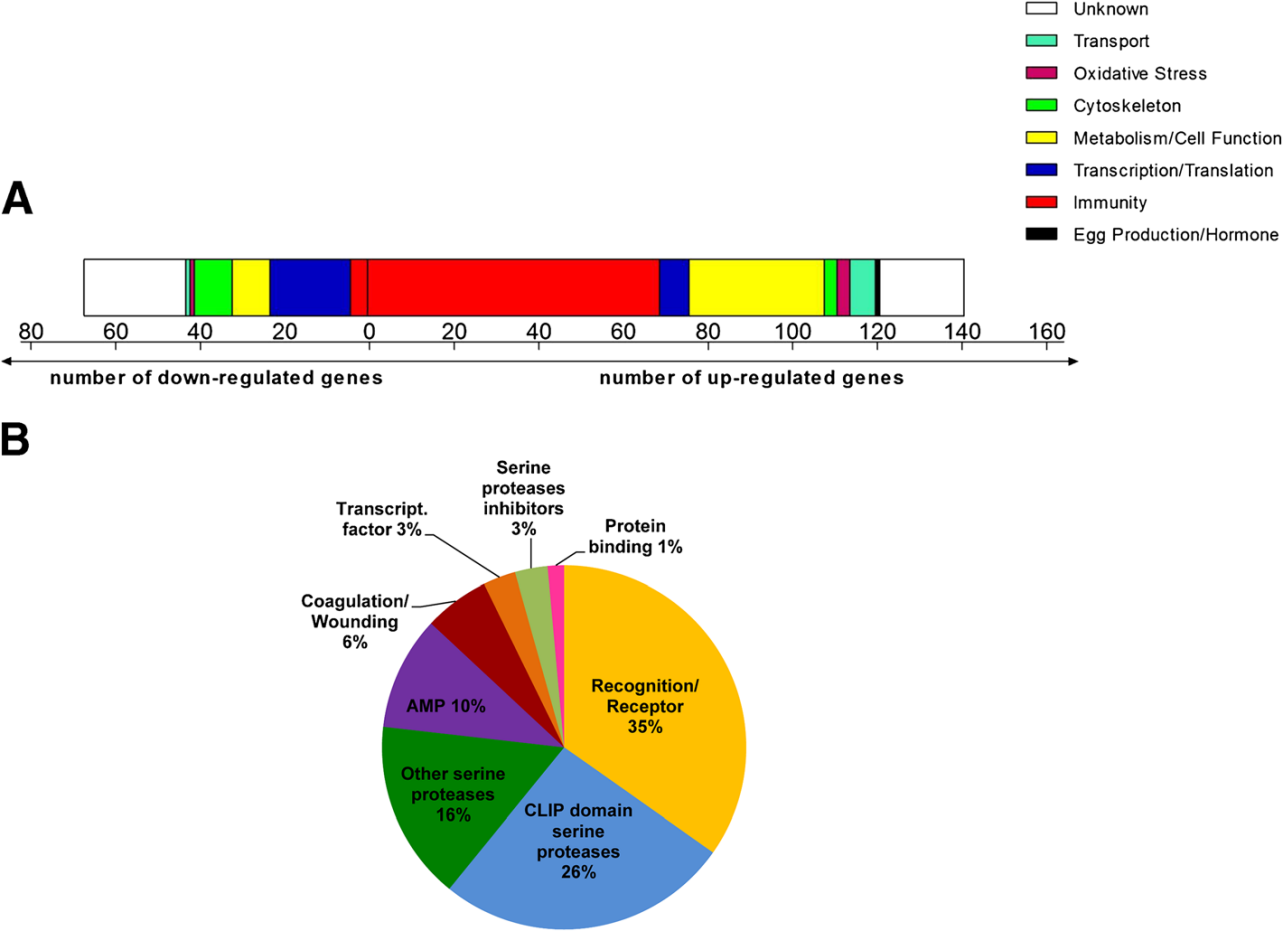
**Functional class distribution of genes regulated by hemozoin. (A)** Coloured sections correspond to the number of genes either up- or down-regulated in the fat body 24 h after female mosquitoes were injected with PBS or 200 μg/ml sHz. **(B)** Pie chart shows subclasses distribution in immunity related up-regulated genes.

To provide further insight into the mechanism underlying the mosquitoes’ decreased susceptibility to *P. berghei* upon treatment with hemozoin, microarray transcription analysis was performed to assess gene expression response of female mosquitoes to injection with 200 μg/ml sHz. Fat bodies were used, as it would be informative of the response to sHz challenge, being the main source of circulating immune related components. Differential expression associated with treatment was observed in a total of 208 genes, from which 141 were up-regulated and 67 down-regulated (Figure 2A and Additional file 4).

The functional class of genes most represented were immune response related genes, comprising 35% of the differentially expressed total genes. From this, 95% were positively induced with sHz treatment compared to control, indicating a robust general activation of the immune system. The majority of down-regulated genes (28%) were involved in transcription and translational functions. Of the up-regulated genes, the most represented class (49%) was immunity, followed by metabolism/cell function (23%) (Figure 2A), which indicates that other processes may be involved in the physiological immune response. Immune up-regulated genes are outlined in Table 1.

**Table 1:** **Immunity related genes differentially induced in the fat body following sHz treatment**

**Gene ID**	**Gene name**	**Lower bound**
**Recognition/Receptor**		
AGAP005848	FBN50	3.46
AGAP009916	Q5XLG8_ANOGA	2.37
AGAP010816	TEP3	2.34
AGAP005335	CTL4	2.27
AGAP005334	CTLMA2	2.08
AGAP006348	LRIM1	1.90
AGAP007039	LRIM4	1.61
AGAP000999	TOLL5A	1.59
AGAP007033	APL1C	1.53
AGAP008364	TEP15	1.52
AGAP010812	TEP4	1.47
AGAP011197	FBN9	1.46
AGAP010772	FBN18	1.37
AGAP010815	TEP1	1.36
AGAP010832	TEP19	1.36
AGAP007045	LRIM15	1.27
AGAP011871		1.27
AGAP005625	SCRASP1	1.26
AGAP008654	TEP12	1.26
AGAP011230	FBN10	1.25
AGAP006644		1.22
AGAP008368	TEP14	1.22
AGAP000871	CTL1	1.20
AGAP010350		1.20
**CLIP domain serine proteases**		
AGAP003689	CLIPC7	4.16
AGAP012037	CLIPB20	3.14
AGAP009844	CLIPB15	2.97
AGAP003252	CLIPB6	2.41
AGAP011781	CLIPA12	2.25
AGAP011790	CLIPA2	2.04
AGAP011780	CLIPA4	1.98
AGAP010833	CLIPB14	1.98
AGAP011792	CLIPA7	1.97
AGAP011791	CLIPA1	1.90
AGAP011788	CLIPA14	1.73
AGAP001648	CLIPB17	1.65
AGAP003249	CLIPB3	1.64
AGAP011789	CLIPA6	1.60
AGAP010731	CLIPA8	1.56
AGAP003057	CLIPB8	1.28
AGAP004855	CLIPB13	1.27
AGAP004148	CLIPB5	1.26
**Other serine proteases**		
AGAP003960		3.26
AGAP003627		2.89
AGAP003248		2.47
AGAP010730		2.33
AGAP007043		1.89
AGAP012614		1.69
AGAP000290		1.64
AGAP008861		1.36
AGAP003626		1.28
AGAP009849		1.25
AGAP013252		1.22
**Antimicrobial peptides (AMP)**		
AGAP006722	CEC4	1.86
AGAP000693	CEC1	1.48
AGAP001508	IRSP3	1.38
AGAP007385	LYSC4	1.35
AGAP011294	DEF1	1.30
AGAP007347	LYSC1	1.22
AGAP007344	LYSC8	1.21
**Coagulation/Wounding**		
AGAP010934		1.81
AGAP011034		1.46
AGAP009098	TGM98	1.36
AGAP011765		1.25
**Serine proteases inhibitors**		
AGAP009213	SRPN16	1.32
AGAP006911	SRPN2	1.29
**Transcription factor**		
AGAP006747	REL2	1.54
AGAP012711		1.48
**Protein binding**		
AGAP009546		1.24

In *A. gambiae*, it has been shown that malaria infection induces a large number of immune effector genes, which form an important line of defence against *Plasmodium* parasites. In our microarray screen, sHz treatment induced several PRRs, which have been associated with control of *Plasmodium* infection in the mosquito (Figure 2B and Table 1). Among them are some potent immune factors, as TEP1 (thioester-containing protein 1), APL1 (*Anopheles Plasmodium*-responsive leucine-rich repeat 1) and FBN9 (fibrinogen immunolectin 9) (Table 1). Evidence from observations in vertebrates and co-localization at parasite surface led Garver *et al*. [23] to speculate that a lectin complement-like pathway mechanism, in which TEP1 and FBN9 cooperate to destroy pathogens, may exist in mosquitoes as well. Our data showed that these two genes presented similar expressions (Table 1). APL1C and LRIM1 (leucine-rich repeat immune protein 1), another two PRR members of the complement-like pathway in *A. gambiae* [24,25], showed similar expressions in our microarray analysis (Table 1). In addition to the interaction with TEP1, the LRIM1/APL1C complex interacts with the mature forms of other proteins such as TEP3 and TEP4 [25], both also up-regulated after sHz treatment (Table 1). The basal expression of TEP1 and LRIM1 is representative of the pre-invasion phase [26], which is in accordance with our results for the induction of these genes in the fat body before parasite invasion. Of note, these genes are believed to be hemocyte-specific, which shows how fat bodies can also comprise other cellular types besides fat body cells, namely hemocytes that are in part sessile and attached to tissues. Other members of the TEP, FBN and LRIM families were up-regulated as well in our screen.

Serine proteases are present in the hemolymph, where they can rapidly activate immune pathways in response to pathogen detection [27], playing as positive and negative regulators. We have observed a very strong stimulation of CLIP domain and other serine proteases in our microarray results, comprising 42% of the total number of up-regulated immune related genes (Figure 2B). However, some of these genes have been classified as agonists of *P. berghei* infection. CLIPA2, CLIPA7, receptors CTL4 (C-type lectin 4) and CTLMA2 (C-type lectin mannose binding 2) and SRPN2 (serpin 2), are all inhibitors of parasite melanization [28-31], hence playing a role in parasite protection. This suggests that melanization is not triggered by sHz treatment, and is not the effector mechanism that confers protection against *P. berghei* observed in this study, explaining why we could not observe any melanized oocysts in the sHz-treated mosquitoes.

*CEC1* and *DEF1*, two AMPs induced by sHz in our microarray screen (Table 1), belong to the most important AMP families, defensins and cecropins [32]. These AMP families have been characterized in *A. gambiae,* where they are produced by the fat body and hemocytes, being secreted into the hemolymph upon immune challenge [33,34]. Ectopic overexpression of CEC1 was shown to increase *A. gambiae* resistance to *P. berghei* [35]. A few immune up-regulated genes related with coagulation/wounding were represented in our microarray results as well.

We observed an increased expression of REL2 in our microarray analysis. This observation has drawn our attention to the fact that many induced immune related genes in this screen have been described by different authors as regulated by this NF-κB transcription factor. Indeed *FBN9, TEP1, TEP4, LRIM1*, *CLIPB14* and *AGAP003960* have all been reported to be REL2-regulated/-partly regulated target genes of the Imd pathway [36-41], which prompted us to hypothesize that the up-regulation of fat body immunity genes following sHz treatment may be affected by this transcription factor expression.

To validate the robustness of the microarray results, ten genes were analysed by real-time qRT-PCR. The two assays showed a high degree of correlation (Additional file 5).

### REL2-F, rather than REL2-S or REL1, is induced by hemozoin in *Anopheles gambiae* cells

Pathogen recognition in *A. gambiae* is followed by signal transduction through Toll and Imd pathways that activate NF-κB transcription factors REL1 and REL2 respectively. These factors translocate to the nucleus to initiate the transcription of many effector genes. Their dependent transcription has been shown to be particularly critical to the mosquito ability to manage infection with *P. berghei* and *P. falciparum* [23,26,36-38,42,43].

Through alternative splicing, two isoforms of REL2 are present in *Anopheles*: REL2-F (a full-length form) and REL2-S, lacking the inhibitory ankyrin repeats and death domain present in REL2-F. Both transcripts are expressed constitutively throughout *A. gambiae*, as well as in cultured cell lines [36,37].

**Figure 3:**
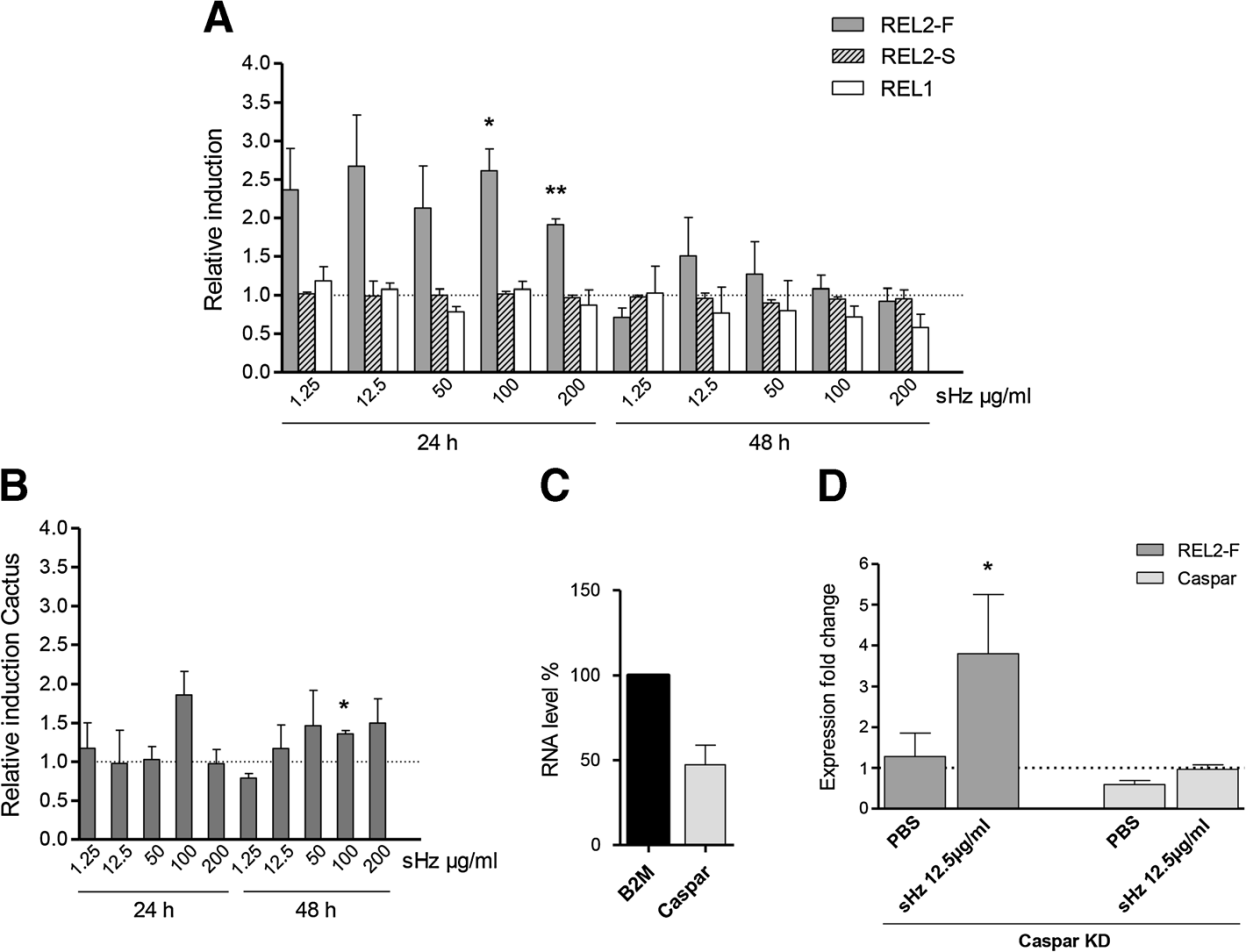
**Transcription factor expression in cells after hemozoin treatment. Combined effect of** ***caspar*** **silencing and sHz treatment.** *A. gambiae* Sua 5.1^*^ cells were stimulated with different concentrations of sHz and the expression of **(A)** REL2-F, REL2-S and REL1 **(B)** REL1’s negative regulator Cactus was measured after 24 h and 48 h. Relative induction fold change was determined by qRT-PCR comparison to PBS-treated controls. **(C)** Cells were silenced using dsRNA specific for *caspar* or control-dsRNA against *β2M*. **(D)** Following silencing, cells were treated with PBS or 12.5 μg/ml sHz. Graph represents expression of REL2-F and Caspar in silenced cells with and without sHz. Relative expressions were measured by qRT-PCR comparison to *β2M*-silenced control. **(A-D)** Values represent the mean and SD of three independent experiments. Differences and *P*-values: *(*p* < 0.05), **(*p* < 0.01), using the *Student t test*.

Based on previous data that confirmed that sHz could function as a signal in mosquito cells [13], we used a cell-based approach to explore *in vitro* the suggestion that hemozoin’s triggering of pathways and immune defence mechanisms verified in this study is due to the activation of REL2. *A. gambiae* hemocyte-like Sua 5.1^*^ cells were stimulated with a range of sHz concentrations, from 1.25 μg/ml to 200 μg/ml, comparable to the ones used in mosquito cells- and murine macrophages-stimulation assays *in vitro* [13,14]. Along with the fat body and epithelial tissues, hemocytes are a source of AMPs production. The levels of REL2-F and REL2-S expression induction were analysed by qRT-PCR 24 hours (24 h) after sHz stimulation, the same time-point used in the microarray experiment, and at 48 h post sHz treatment. At 24 h after treatment, all sHz concentrations used to stimulate Sua 5.1^*^ cells induced REL2-F average expression from 1.91- to 2.67-fold relative to PBS-control, with significant (*p* = 0.0165 and *p* = 0.0043) up-regulation shown at 100 μg/ml and 200 μg/ml sHz respectively. REL2-F induction at 48 h was observed with 12.5 μg/ml and 50 μg/ml sHz, but not significantly and at a much lower level than at 24 h. REL2-S expression was unaltered at both time points and for all concentrations (Figure 3A).

To examine if other factors would respond as well to hemozoin stimulus, we measured the expression of REL1 after sHz treatment. Notwithstanding a subtle induction observed after treatment with the lowest sHz concentration, REL1 expression was mostly unaltered at 24 h and down-regulated at 48 h post sHz treatment (Figure 3A). When the relative induction of REL1’s negative regulator Cactus was measured, a general induction was observed, particularly after 48 h and for almost every concentration, which was significant (*p* = 0.0180) when cells were stimulated with 100 μg/ml sHz (Figure 3B). Cactus induction opposed REL1’s low expression, thus supporting the idea that REL1 is indeed down-regulated by hemozoin.

These findings seem to confirm that hemozoin induces the expression of REL2 and this induction involves its full-length form, REL2-F (rather than the short form REL2-S), as we had observed in our microarray data (the up-regulated REL2 probe set binds to the ankyrin domain characteristic of REL2-F). Moreover, this induction is maximal at 24 h after sHz stimulation. However, at 48 h post sHz treatment an increase in REL2-F expression was still detectable, suggesting that REL2-F gene expression is sustainable for at least 48 hours. REL1 transcription factor was not positively stimulated by hemozoin, which was confirmed by the consistent activation of its negative regulator Cactus upon sHz treatment.

### Hemozoin further up-regulates REL2-F upon caspar silencing

Silencing of target genes in *A. gambiae* and other *Anopheles* species has proved to change the mosquito susceptibility to *Plasmodium* infection. Particularly, knock-down of the negative regulators of the Toll and Imd pathways, *cactus* and *caspar* respectively, has shown to control malaria infection [23,26,43].

To strengthen our fat body transcriptional profiling and hemocyte-like cell-based observations that suggested hemozoin is an activator of the REL2 pathway, we next investigated, by RNAi analysis, the effect of *caspar* silencing on REL2-F induction in Sua 5.1^*^ cells treated with hemozoin. A preceding experiment to test which dsRNA concentration would best knockdown *caspar* was performed with this gene silenced from 24 h to 72 h. dsRNA concentrations varied from 5 to 22.5 μg/ml, according to the ones used in other studies for gene silencing by RNAi in *A. gambiae* cells [37,44]. The best silencing efficiency was achieved with the highest dsRNA concentration tested and an incubation period of 48 h (data not shown).

Cells were treated with *caspar-*targeting dsRNA or dsRNA targeting *β2M* as control, at the highest tested concentration. One day following silencing, control-PBS or 12.5 μg/ml sHz was added for 24 h, the concentration and time point we observed above (Figure 3A) as having caused the highest REL2-F induction. The levels of REL2-F were assessed by qRT-PCR 48 h post silencing (24 h post sHz treatment).

The average silencing efficiency obtained was 52.7% (Figure 3C). *caspar* silencing up-regulated REL2-F relative expression and when silenced cells were treated with sHz, REL2-F was further induced up to an average significant (*p* = 0.0247) level of 3.79-fold change compared to control (Figure 3D). Caspar levels of expression were also measured. Following silencing of the gene, Caspar was down-regulated, as expected. Adding sHz has slightly increased the relative expression of this gene. Although *caspar* had been silenced, it was not completely knocked-down and this slight increase of its expression following sHz treatment might reflect a counterbalanced response to the high levels of REL2-F expression. In parallel, we designed a *cactus*-silencing/REL1 experiment but, as the silencing efficiency obtained was below 50%, results are not shown here. However, it is worth mentioning that, although REL1 expression was up-regulated after *cactus* silencing, it did not change when sHz was added. In conclusion, the results obtained through dsRNA-based silencing of its negative regulator, further indicate that REL2-F transcription factor is indeed efficiently induced by hemozoin.

### Hemozoin up-regulates REL2-F and other immune related genes *in vivo* before and after *Plasmodium berghei* infection

**Figure 4:**
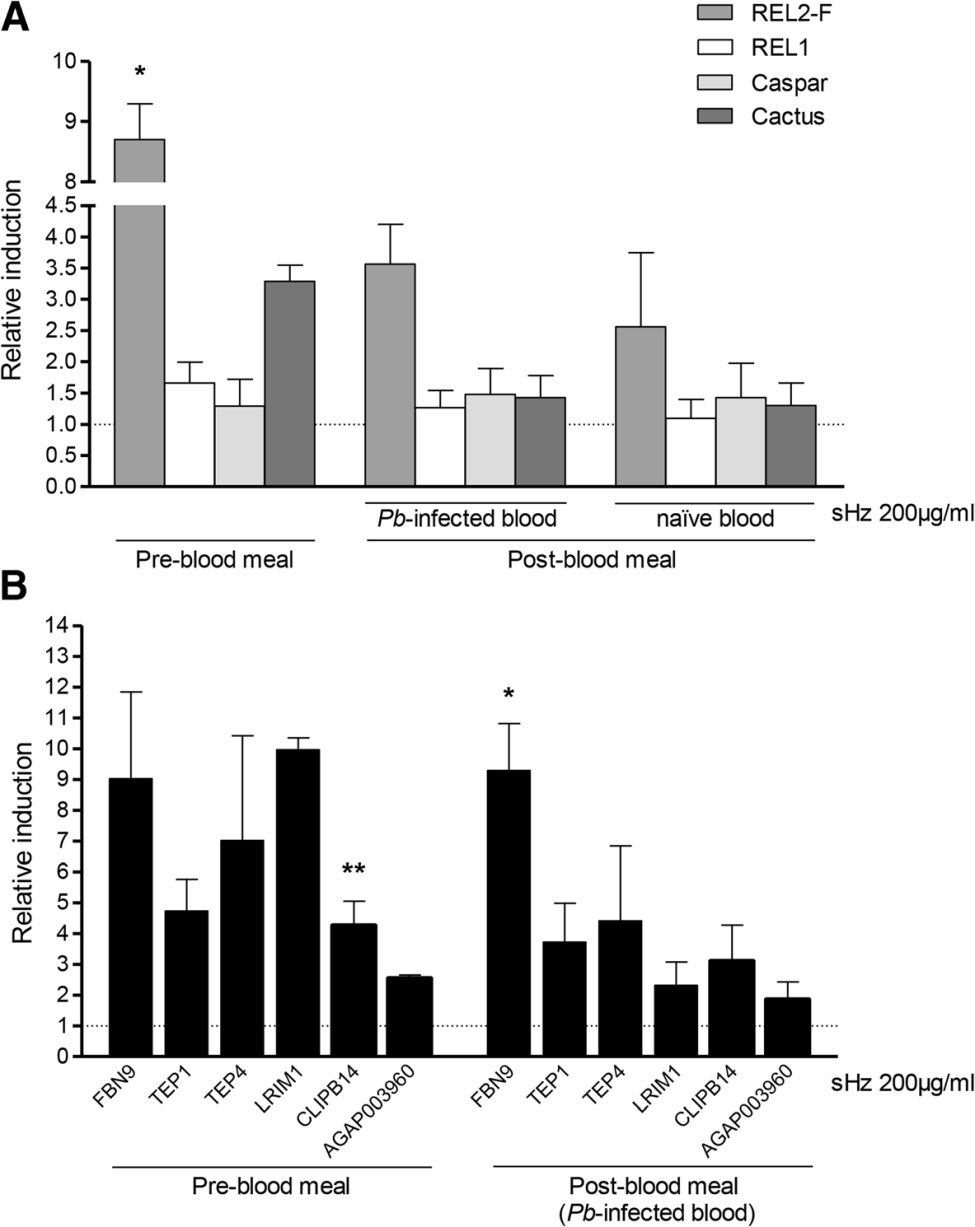
**Effect of hemozoin** ***in vivo*** **before and after the blood meal.** Female *A. gambiae* were treated with 200 μg/ml sHz and fed with *P. berghei-*infected or naïve blood 24 h later. The expression of **(A)** REL2-F, REL1, Caspar, Cactus and **(B)** six immune related genes transcription was measured in the fat body before and after blood ingestion. Relative induction fold change was determined by qRT-PCR comparison to PBS-treated controls. Values represent the mean and SD of three independent experiments. Differences and *P* values: *(*p* < 0.05), **(*p* < 0.01), using the *Student t test*.

Having shown that hemozoin’s REL2-F activation lasts for 48 h at least, we then analysed *in vivo* the expression of this and other factors at different time points, following stimulation with 200 μg/ml sHz, the same concentration used in the microarray experiment and which provoked a significant REL2-F up-regulation in the cellular assay *in vitro*. The levels of expression induced were measured by qRT-PCR in the fat bodies of female mosquitoes 24 h after sHz stimulation (immediately before blood ingestion), as well as 48 h after sHz stimulation, 24 h after the mosquitoes were provided with a blood meal. As shown in Figure 4A, at 24 h after treatment, before the blood meal, hemozoin induced REL2-F average expression up to a significantly (*p* = 0.0253) high level of 8.71-fold change relative to PBS control. After the blood meal, REL2-F was still markedly up-regulated, but at a lower level than before the blood meal. The expression levels of REL1 and the negative regulators Caspar and Cactus were mostly consistent throughout the assay, fluctuating from unaltered to slightly up-regulated upon sHz stimulation compared to control, with the exception of a higher but not significant Cactus expression level pre-blood meal.

Mosquitoes were fed with either *P. berghei*-infected blood or noninfected (naïve) blood as control. The decrease in REL2-F expression levels after the blood meal is most probably due to the fact that they were measured 48 h after sHz stimulation, rather than *P. berghei* infection altering the expression levels, as the same pattern of REL2-F decreased induction was observed both after the *P. berghei*-infected and the naïve blood meals. These results agreed with the microarray outcome and confirmed what we had observed in cells: REL2-F activation by sHz is maximal at 24 h, however 48 h post sHz stimulation REL2-F is still induced. This shows that REL2-F activation by sHz is a broad mechanism, observed both *in vitro* and *in vivo*.

Similarly, the relative expression levels of six important immune related genes (*FBN9, TEP1, TEP4, LRIM1*, *CLIPB14* and *AGAP003960*) up-regulated in the microarray analysis, were measured in the female fat body before and after the *P. berghei*-infected blood meal. In agreement with REL2-F’s expression, these genes were induced by sHz before the blood meal, as well as after the blood meal though at a lower level for most of them (Figure 4B). Of note, it has been said that the mosquito basal immunity level (before the mosquito encounters the parasite) is a key factor for parasite control [26], which reinforces the importance of these genes’ up-regulation in the pre-invasion phase. A random batch of *P. berghei*-infected mosquitoes was left to check the success of infection by screening for the presence of oocysts (Additional file 6), with the pattern of infection reduction described in Figure 1 being confirmed.

### REL2 mediates the hemozoin effect

**Figure 5:**
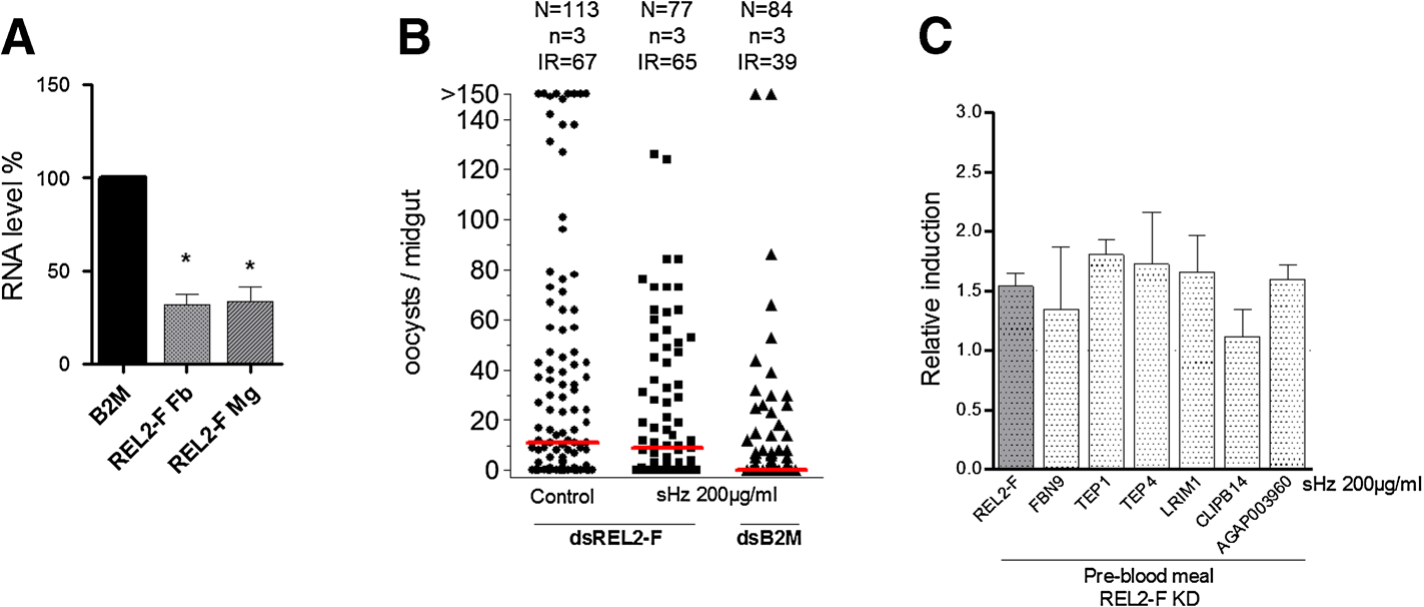
**REL2-F silencing suspends the hemozoin effect.** Female mosquitoes were silenced using dsRNA specific for *REL2-F* or control-dsRNA against *β2M.* **(A)** Silencing efficiency was measured both at the fat body (Fb) and the midgut (Mg). Differences and *P* values: *(*p* < 0.05), using the *Student t test*. **(B)** Silenced mosquitoes were treated with sHz or PBS-control and infected with *P. berghei*; oocysts were counted 8 days post-infection. Horizontal red bars represent the median. N, number of female mosquitoes; n, number of independent experiments; IR, infection rate (%). **(C)** Relative induction fold change of REL2-F and six other immune related genes measured in the fat body following REL2-F silencing and subsequent sHz treatment, determined by qRT-PCR comparison to PBS-treated controls.

We have demonstrated above how hemozoin stimulates REL2-F both *in vivo* (Table 1, Additional file 4, Figure 4A) and *in vitro* (Figure 3A), and shown this molecule’s impact in the *Plasmodium* infection outcome, reducing both infection rate and intensity (Additional file 2, Figure 1). To explore whether REL2 mediates hemozoin effect, we next investigated whether sHz treatment still had an effect on parasite numbers in mosquitoes silenced for REL2-F. We used dsRNA as in [36], which is specific to target the ankyrin domain of REL2, hence should only affect REL2-F. Silencing efficiency was measured 4 days after dsRNA injection. REL2-F was efficiently silenced, at both the mosquito fat body (68.1%) and midgut (66.3%) (Figure 5A). Note that this is not a measure of available protein, which would only be possible through western blot analysis of dsRNA-injected mosquitoes. Silenced mosquitoes were treated either with control-PBS or 200 μg/ml sHz and fed with *P. berghei*-infected blood 24 h later. Oocysts from the midguts of the three groups of mosquitoes were counted after 8 days (Figure 5B). Hemozoin did not affect the infection rate in *REL2-F*-silenced mosquitoes, as the percentage of infected mosquitoes is equivalent for both control-PBS treated and sHz-treated mosquitoes, in a total of 190 *REL2-F*-silenced *A. gambiae* females. In contrast, the infection rate in control-dsβ2M-injected sHz treated mosquitoes (39%) was lower when compared to *REL2-F*-silenced sHz treated mosquitoes (65%), in a total of 161 *A. gambiae* females.

The statistical modelling using an approach similar to the one used above, revealed a best fitting to ZINB model (Additional file 3: Table S3). Results show that the effect of the different experiments was not significant. In terms of gene silencing and sHz treatment, comparing dsREL2-F + sHz with dsREL2-F + PBS groups, a significant difference exists in the number of oocysts >0 but no significant difference was found in the number of zeros (non-infected mosquitoes). Comparing dsREL2-F + sHz with dsβ2M + sHz, the model shows a significant increase in the number of zeros (non-infected mosquitoes) in the dsβ2M + sHz mosquitoes and no significant differences in the number of oocysts >0.

Taken together, these results demonstrate that the effect of hemozoin is suspended when REL2-F is knocked-down. The levels of infection, which are increased with *REL2-F* silencing compared to control-silencing, due to more susceptibility to infection in REL2-F-depleted mosquitoes, as observed by other authors both for *P. berghei* and *P. falciparum* [36,43], do not show reduction with sHz’s treatment when compared to control PBS treatment, in contrast with what has been observed in Figure 1 for non-silenced mosquitoes.

The expression levels of six immune related genes reported by some authors as REL2-regulated and which were induced by sHz before the blood meal (Figure 4B), were also analysed, to evaluate these genes response to sHz following REL2-F silencing. As can be seen in Figure 5C, the average relative expression levels of all genes following sHz treatment, was markedly impaired in the REL2-F-silenced fat bodies (levels from 1.12- to 1.80-fold change) when compared to the non-silenced ones (levels from 2.54- to 9.96-fold change, Figure 4B), indicating that when REL2-F is silenced, sHz modulation of these genes expression is almost ineffective.

Altogether, the results obtained (Figure 5) suggest that the more efficient antiparasitic response observed upon sHz treatment is mediated by REL2, through activation of the expression of downstream genes, including those involved in parasite elimination.

## Discussion

Several experimental examples have demonstrated that *Anopheles* mosquitoes are able to mount an efficient immune response against *Plasmodium* infection, which is responsible for big parasite losses during parasite development. Even so, natural immunity is not enough to totally suppress mosquito infection. In previous works, we showed that the *Anopheles* immune system can be modulated by outside factors such as antimalarial drug chloroquine [45,46], and further we observed that synthetic CpG ODNs can induce protective responses in *Anopheles* against *Plasmodium* [47].

The results of this work demonstrate that the parasite’s by-product hemozoin has a noticeable effect in the *Plasmodium* infection outcome, impairing both infection rate and intensity.

Our transcription analysis established that hemozoin can act as an immunomodulatory molecule for the mosquito. The most remarkable result obtained from our microarray screen was the differential up-regulation of the NF-κB factor REL2 upon hemozoin stimulation. In the only study testing hemozoin’s effect on *Anopheles* published so far [13], authors showed hemozoin activates TAK1 (TGF-beta activated kinase 1) and proposed it may initiate the response leading to REL2 activation. However, no further work has been reported to elucidate the interaction between the parasite’s by-product and *Anopheles* immunity, until the findings we present here.

Several studies before ours have linked *A. gambiae* Imd pathway/REL2 to the defence against *P. berghei* and in that way established a role for this pathway in anti-*Plasmodium* response [23,36,41,43]. In a recent study where both Imd and Toll pathways’ negative regulators, *caspar* and *cactus,* were silenced, *caspar* silencing influenced transcription of fewer genes, but a considerable number of regulated genes had immunity-related functions [23]. This is in concordance with our findings, where the activation of the Imd/REL2 pathway by hemozoin led to the transcription of a high number of immunity genes (35% of the total differentially expressed genes) (Figure 2, Table 1), that might have contributed to the malaria resistant phenotype observed in this study.

Recent studies have demonstrated that the defence mechanisms mounted by the mosquito against bacterial invasion incite the activation of genes associated with the immune response against *Plasmodium* [40,48-51] and, in most cases, the consequent elimination of a large number of parasites. In *A. gambiae*, REL2 was shown to be involved in the signalling pathway activated by PGRPLC (peptidoglycan recognition protein lc) following bacterial infections [48]. In a forthcoming study, it will be of great interest to elucidate the interactions between hemozoin stimulation, bacteria initiation and Imd/REL2 pathway activation of the response against *Plasmodium* in *Anopheles*.

Our microarray results identified the up-regulated REL2 probe set as the specific one for the full-length isoform of REL2, REL2-F. The use of primers specific for each isoform, in further experiments*,* enabled us to confirm that REL2-F was up-regulated when mosquitoes and cells were treated with sHz. In the absence of immune stimulation, REL2 exists in the two variants: REL2-S, that is constitutively active, and REL2-F, that is inactive until immune stimulus. Imd pathway activation stimulates cleavage of the inhibitory ankyrin terminal domain of REL2-F, exposing it to nuclear translocation and subsequent transcription initiation [39]. When Meister and co-workers [36] silenced REL2 in *A. gambiae* mosquitoes and measured infection by *P. berghei* afterwards, they obtained statistically similar infection results when silencing REL2-F only and both forms of REL2 together concluding that REL2-F, instead of REL2-S, is implicated in this reaction. Moreover, the results of the mentioned study [36], as well as other studies [26,37] in which *A. gambiae* REL2 gene was silenced, indicate that, at least with respect to REL2, the RNAi gene silencing is a good prediction of the likely decrease in REL2 protein. In agreement with what we observed in our work (Figure 5C), dsRNA-based silencing of REL2 gene in these studies down-regulated the expression of immune factors, including PRRs and AMPs. Having shown increased expression of REL2 upon sHz stimulation both in the fat body and in hemocyte-like cells, we wanted to make sure this was the main transcription factor activated by hemozoin; REL1 expression levels revealed that indeed the Toll pathway didn’t seem to be meaningfully activated by sHz.

By silencing *caspar,* we were able to further confirm hemozoin’s REL2-F activation (Figure 3D). Note that Caspar is thought to specifically target the REL2-F branch of the Imd/REL2 immune pathway, as only the full-length isoform REL2-F, contrary to the short one REL2-S, has inhibitory domains that must be cleaved for activation [23]. Hence, the results we obtained upon *caspar* silencing come in concordance with our suggestion that REL2-F and not REL2-S is the isoform triggered by hemozoin. Importantly, other studies have shown that transient activation of either REL1 or REL2 through gene silencing of their negative regulators impairs the success of *Plasmodium* infection, by activation of an immune response before pathogen challenge [23,26]. But when the fitness cost of both manipulations was measured, silencing of *cactus* in laboratory conditions affected *A. gambiae* longevity and fecundity, while *caspar* silencing had no apparent fitness cost. Hence authors concluded that manipulation of the Toll pathway would be unfavourable for the mosquito, probably because this pathway has a more wide-ranging action, while Imd is more immune specific. Overall, these properties of the Imd/REL2 pathway suggest that it could be used in the development of malaria control approaches.

## Conclusions

In our work, we showed that hemozoin treatment increases *Anopheles* resistance to *P. berghei* infection, which was associated with altered transcription profiles of immune-related genes**.** Microarray data and *in vivo* and *in vitro* experiments, indicate that REL2 is regulating the transcription of these genes, following hemozoin stimulation. To explore if REL2 mediates hemozoin’s effect, *A. gambiae* mosquitoes were silenced for the REL2-F gene, revealing that hemozoin’s action is suspended when REL2-F is knocked-down. These findings may well explain our observations and lead us to propose that hemozoin stimulated REL2-F, enabling its translocation to the nucleus, where it further activated several immunity related genes. The activation of these genes contributed to the *P. berghei*-resistant phenotype in *A. gambiae* shown in this study.

In our work, we elucidated for the first time the stimulatory activity of the malarial metabolite hemozoin in the *A. gambiae* vector, while unravelling effector mechanisms of the mosquito protective immune response.

## Additional files

Additional file 1:
**Primers used in qRT-PCR and RNAi assays.** dsRNA primers include the T7 promoter sequence. Additional file 2:
**Infection rate and intensity of** ***Plasmodium berghei*** **in control/sHz injected** ***Anopheles gambiae.*** Data for all experiments using control-PBS and (A) 100 μg/ml sHz or (B) 200 μg/ml sHz. N, number of female mosquitoes per experiment. Additional file 3:
**Coefficients and** ***P*** **values associated to ZINB model and log-likelihood, AIC and summary of Pearson residuals.** (Table S1) 100 μg/ml sHz, (Table S2) 200 μg/ml sHz, (Table S3) Effect of REL2-F silencing. Additional file 4:
**Microarray-derived gene expression in** ***Anopheles gambiae*** **fat body following control-PBS or sHz inoculation.** The list includes all differentially expressed genes. Additional file 5:
**Validation of microarray analysis using qRT-PCR.** Gene expression values for ten genes obtained by microarray plotted against the corresponding averages of three qRT-PCR-derived gene expression values from biological replicates. The Pearson correlation coefficient (*p* = 0.8976) and the best-fit linear-regression analysis (R^2^ = 0.8058) demonstrated a high degree of correlation between gene expression magnitudes determined by each assay. Additional file 6:
**Infection rate and intensity of** ***Plasmodium berghei*** **in control/200 μg/ml sHz injected** ***Anopheles gambiae.*** N, number of female mosquitoes per experiment.
