# Supplementary material for: Hemozoin activates the innate immune system and reduces Plasmodium berghei infection in Anopheles gambiae
Source: Parasit Vectors. 2015 Jan 8;8:12. doi: 10.1186/s13071-014-0619-y (PMC4297457; doi:10.1186/s13071-014-0619-y)
Supplement: Additional file 1: — Primers used in qRT-PCR and RNAi assays. dsRNA primers include the T7 promoter sequence. [file 13071_2014_619_MOESM1_ESM.docx]

| **Gene name** | **Gene ID Ensembl** | **Forward primer (5’-3’)** | **Reverse primer (5’-3’)** |
| --- | --- | --- | --- |
| *CEC1* | AGAP000693 | CAACCCAGAGACCAACCAACCAC | ACTGCCAGCACGACAAAGATGAAG |
| *CLIPC7* | AGAP003689 | GAGATTGAACACTGCCACGA | GTTCCGACACATTCCACCTT |
| *CTL4* | AGAP005335 | TTGAATTGGTTTGATGCCGTGTCCTA | GGCGCTCGTTGGTATCCTTTATTGT |
| *FBN50* | AGAP005848 | ATCACAAGGTTCCGGCTATG | CGTTGGTGTAGGTGAGCAGA |
|  | AGAP006809 | CCCTGTTCACCTTCAAGCAG | GGTCAGCACGGCATCATACT |
| *SRPN2* | AGAP006911 | AGTCTCGAGGGCGCGGTCATTACG | GGGTTTGCCGCGAGTGCCATAGA |
|  | AGAP010056 | CGATGATGGTCCTCGTTTTT | TTCCATCGAGGATTTTCACC |
| *TEP4* | AGAP010812 | AGCACCAGCGCCTTAATCT | CCGCTGATCCAATTTATCGT |
| *DEF1* | AGAP011294 | GGAGAACTATCGGGCCAAG | GATACAGTGAGCGGCACAAA |
| *CLIPA2* | AGAP011790 | AGCCCTTCTGCCCTTCTTAACAAC | CGTCGGTGGTGCGTTCTCTTC |
| *S7* | AGAP010592 | CATTCTGCCCAAACCGATGCGT | CGGGAATACCAGATCCTCCAGG |
| *REL2-F* | AGAP006747 | ACCGATACGGAAAGTGTGCT | GTATCGTTGCGTCGGATTG |
| *REL2-S* | AGAP006747 | ACCGATACGGAAAGTGTGCT | CGGTGCTCCTCGTAATGACT |
| *REL1* | AGAP009515 | GGTCGTGTCGGACATCATCT | TCTCGAAAAAGCGCACCTTA |
| *Cactus* | AGAP007938 | GACGGCTTATCAGCTTGCAC | AACGCACTCGCTCCGTAGTA |
| *Caspar* | AGAP006473 | GGTGGGACTTGTCGTTTGAA | CTTCCATCAGCTTGGACAGC |
| *dsCaspar* | AGAP006473 | TAATACGACTCACTATAGCCGCTTTTCTAAACGCTGTC | TAATACGACTCACTATAGAAACAGGTTGCATGTGTGGA |
| *dsB2M* | NM_009735 | TAATACGACTCACTATAGGGAGAcacccccactgagactgataca | TAATACGACTCACTATAGGGAGA  AATTAGGCCTCTTTGCTTTACCA |
| *dsREL2-F* | AGAP006747 | GAATTAATACGACTCACTATAGGGAGAAATCCGACGCAACGATACG | GAATTAATACGACTCACTATAGGGAGAGACCGCAATGTGAAGGATG |
| *FBN9* | AGAP011197 | CCAAGATGTCGGGCAAGTAT | TTGTGGTACGTCAGCGAGTC |
| *TEP1* | AGAP010815 | CAGATGGTTCGTTTGGTGTG | GCAATGCCGTCAACACATAC |
| *LRIM1* | AGAP006348 | AACGGACAGCAGCCTAAAGC | AGATCAAGCTCCTTTACGTTCCA |
| *CLIPB14* | AGAP010833 | TTCGCGCATTCAACAAGGAG | ATGCCGGCCAAATACCAC |
|  | AGAP003960 | TGGTACATACGGGGTTTGGT | GGTACTGCTCGATCCACCTC |

Additional file 1. Primers used in qRT-PCR and RNAi assays*.*
