# Supplementary material for: Hemozoin activates the innate immune system and reduces Plasmodium berghei infection in Anopheles gambiae
Source: Parasit Vectors. 2015 Jan 8;8:12. doi: 10.1186/s13071-014-0619-y (PMC4297457; doi:10.1186/s13071-014-0619-y)
Supplement: Additional file 2: — Infection rate and intensity of Plasmodium berghei in control/sHz injected Anopheles gambiae. Data for all experiments using control-PBS and (A) 100 μg/ml sHz or (B) 200 μg/ml sHz. N, number of female mosquitoes per experiment. [file 13071_2014_619_MOESM2_ESM.docx]

Additional file 2. Infection rate and intensity of *P. berghei* in control / sHz injected *A. gambiae.*

A.

| **100 µg/ml sHz** | | | | | | | | |
| --- | --- | --- | --- | --- | --- | --- | --- | --- |
|  | Experiment 1 | | Experiment 2 | | Experiment 3 | | Experiment 4 | |
| N | 28 | | 65 | | 91 | | 37 | |
|  | PBS | sHz | PBS | sHz | PBS | sHz | PBS | sHz |
| Infection rate (%) | 63.2 | 44.4 | 44.7 | 25.9 | 72.5 | 64.7 | 53.8 | 41.7 |
| Infection intensity | 22.6 | 16.8 | 56.1 | 40.9 | 63.5 | 56.7 | 21.7 | 20.4 |

B.

| **200 µg/ml sHz** | | | | | | | | |
| --- | --- | --- | --- | --- | --- | --- | --- | --- |
|  | Experiment 1 | | Experiment 2 | | Experiment 3 | | Experiment 4 | |
| N | 36 | | 53 | | 48 | | 56 | |
|  | PBS | sHz | PBS | sHz | PBS | sHz | PBS | sHz |
| Infection rate (%) | 81.3 | 33.3 | 49.1 | 31.0 | 53.0 | 41.7 | 84.6 | 70.0 |
| Infection intensity | 68.7 | 19.3 | 70.3 | 21.3 | 40.1 | 38.2 | 98.8 | 60.6 |
