# Supplementary material for: Hemozoin activates the innate immune system and reduces Plasmodium berghei infection in Anopheles gambiae
Source: Parasit Vectors. 2015 Jan 8;8:12. doi: 10.1186/s13071-014-0619-y (PMC4297457; doi:10.1186/s13071-014-0619-y)
Supplement: Additional file 3: — Coefficients and P values associated to ZINB model and log-likelihood, AIC and summary of Pearson residuals. (Table S1) 100 μg/ml sHz, (Table S2) 200 μg/ml sHz, (Table S3) Effect of REL2-F silencing. [file 13071_2014_619_MOESM3_ESM.docx]

Additional file 3. Coefficients and *P* values associated to ZINB model and log-likelihood, AIC and summary of Pearson residuals.

Table S1. **100 μg/ml sHz.**

|  | ZINB  coefficients (p-values) | |
| --- | --- | --- |
|  | Count model (b) | Zero-inflation (c) |
| Intercept | 2.90682 (<0.001*) | -1.0091 (0.088) |
| Experiment 1 | (a) | (a) |
| Experiment 2 | 0.97058 (0.030*) | 1.0921 (0.075) |
| Experiment 3 | 1.17093 (0.003*) | -0.5309 (0.395) |
| Experiment 4 | 0.05026 (0.919) | 0.3463 (0.613) |
| Treatment | -0.16732 (0.532) | 0.6875 (0.064) |
| **Log(theta)** | -0.69223 (0.006*) | |
| **Log-Likelihood** | -699.3; degree of freedom=11  1420.527 | |
| **AIC** |  |  |
| **Pearson residuals** | Min 1Q Median 3Q Max  -0.612963 -0.487194 -0.378113 -0.002069 6.378019 | |

1. Reference category
2. Oocyst count
3. non-infected mosquitos

Table S2. **200 μg/ml sHz.**

|  | ZINB  coefficients (p-values) | |
| --- | --- | --- |
|  | Count model (b) | Zero-inflation (c) |
| Intercept | 4.0779 (<0.001*) | -1.2882(0.006*) |
| Experiment 1 | (a) | (a) |
| Experiment 2 | -0.1124 (0.776) | 1.0402 (0.052) |
| Experiment 3 | -0.1634 (0.693) | 0.7866 (0.150) |
| Experiment 4 | 0.5362 (0.132) | -0.8086 (0.189) |
| Treatment | -0.6583 (0.018*) | 0.9541 (0.010*) |
| **Log(theta)** | -0.5584 (0.002*) | |
| **Log-Likelihood** | -665.5; degree of freedom=11  1352.914 | |
| **AIC** |  |  |
| **Pearson residuals** | Min 1Q Median 3Q Max  -0.6906 -0.5149 -0.3669 0.1597 4.3472 | |

1. Reference category
2. Oocyst count
3. non-infected mosquitos

Table S3. **Effect of REL2-F silencing.**

|  | ZINB  coefficients (p-values) | |
| --- | --- | --- |
|  | Count model (b) | Zero-inflation (c) |
| Intercept | 3.2599 (<0.001*) | -1.26142 (<0.001*) |
| Experiment 1 | (a) | (a) |
| Experiment 2 | 0.4080 (0.082) | 0.49214 (0.192) |
| Experiment 3 | 0.2561 (0.240) | 0.61022 (0.082) |
| dsREL2-F+PBS (1) | 0.4617 (0.028 *) | -0.01711 (0.961) |
| dsREL2-F+sHz (2) | (a) | (a) |
| dsβ2M+sHz (3) | -0.0736 (0.778) | 1.21485 (<0.001*) |
| **Log(theta)** | -0.2838 (0.049*) | |
| **Log-Likelihood** | -927.9; degree of freedom=11  1877.841 | |
| **AIC** |  |  |
| **Pearson residuals** | Min 1Q Median 3Q Max  -0.7058 -0.6060 -0.4261 0.1634 8.6681 | |

1. Reference category
2. Oocyst count
3. non-infected mosquitos
