# Supplementary figures and images for: Hemozoin activates the innate immune system and reduces Plasmodium berghei infection in Anopheles gambiae
Source: Parasit Vectors. 2015 Jan 8;8:12. doi: 10.1186/s13071-014-0619-y (PMC4297457; doi:10.1186/s13071-014-0619-y)

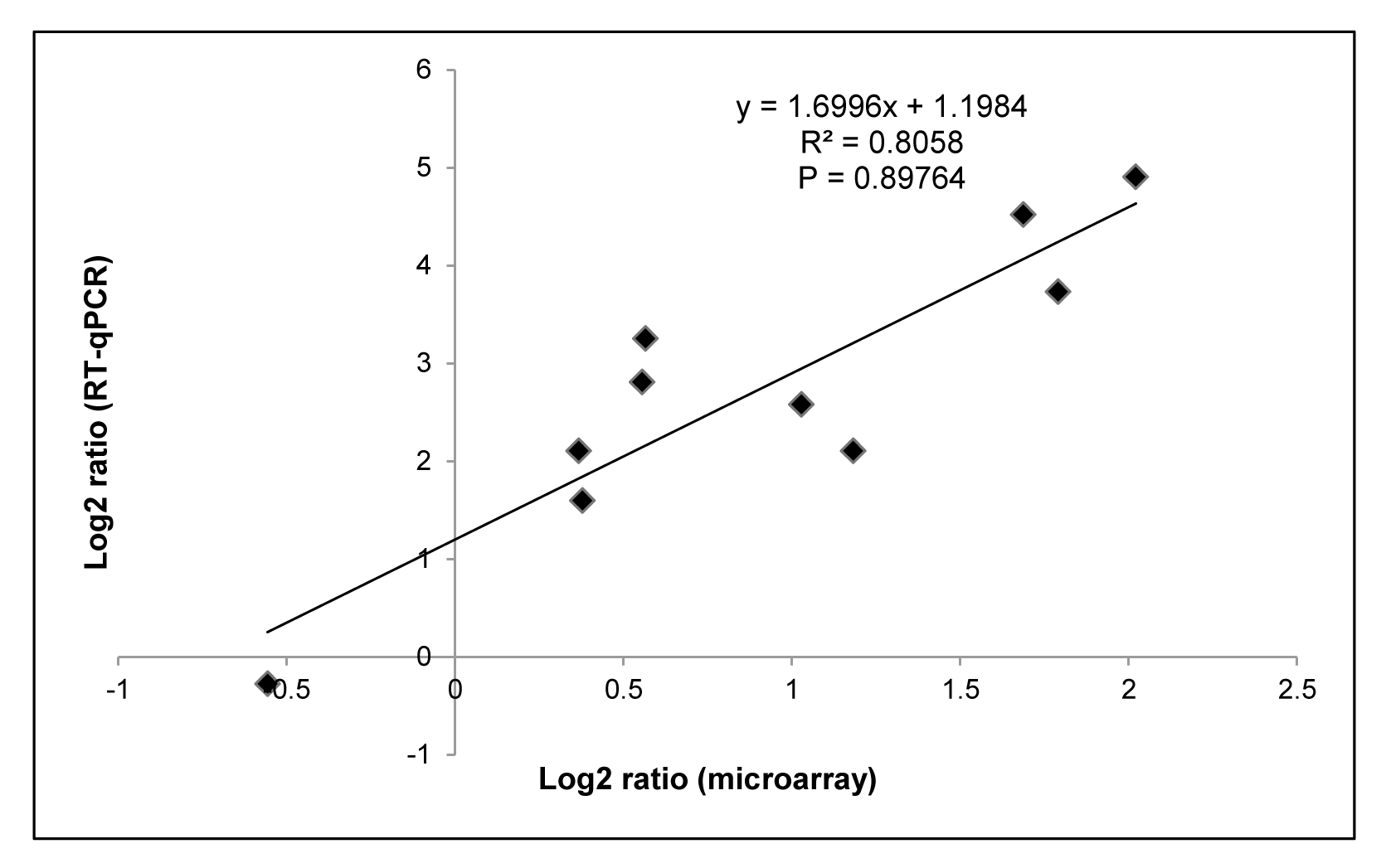

Supplement: Additional file 5: — Validation of microarray analysis using qRT-PCR. Gene expression values for ten genes obtained by microarray plotted against the corresponding averages of three qRT-PCR-derived gene expression values from biological replicates. The Pearson correlation coefficient (p = 0.8976) and the best-fit linear-regression analysis (R2 = 0.8058) demonstrated a high degree of correlation between gene expression magnitudes determined by each assay. [file 13071_2014_619_MOESM5_ESM.tiff]
