# Supplementary material for: Hemozoin activates the innate immune system and reduces Plasmodium berghei infection in Anopheles gambiae
Source: Parasit Vectors. 2015 Jan 8;8:12. doi: 10.1186/s13071-014-0619-y (PMC4297457; doi:10.1186/s13071-014-0619-y)
Supplement: Additional file 6: — Infection rate and intensity of Plasmodium berghei in control/200 μg/ml sHz injected Anopheles gambiae. N, number of female mosquitoes per experiment. [file 13071_2014_619_MOESM6_ESM.docx]

Additional file 6. Infection rate and intensity of *P. berghei* in control / 200 μg/ml sHz injected

*A. gambiae.*

| **200 µg/ml sHz** | | | | | | |
| --- | --- | --- | --- | --- | --- | --- |
|  | Experiment 1 | | Experiment 2 | | Experiment 3 | |
| N | 37 | | 40 | | 44 | |
|  | PBS | sHz | PBS | sHz | PBS | sHz |
| Infection rate (%) | 94.1 | 80.0 | 83.3 | 70.0 | 96.4 | 81.3 |
| Infection intensity | 81.9 | 60.1 | 36.8 | 27.4 | 109.5 | 25.0 |
